# Factors affecting anticipatory grief of family carers supporting people living with Motor Neurone disease: the impact of disease symptomatology

**DOI:** 10.1080/21678421.2024.2359559

**Published:** 2024-05-30

**Authors:** Ana Paula Trucco, Mizanur Khondoker, Naoko Kishita, Tamara Backhouse, Helen Copsey, Eneida Mioshi

**Affiliations:** 1 School of Health Sciences, University of East Anglia, Norwich, UK; 2 Norwich Medical School, University of East Anglia, Norwich, UK, and; 3 Norfolk and Norwich University Hospital Foundation Trust, Norwich, UK

**Keywords:** Anticipatory grief, factors, carers, motor neurone disease, behavioral changes, disease severity

## Abstract

**Objective:**

To investigate the effect of carer- and disease-related factors on anticipatory grief (AG) in family carers supporting people living with Motor Neurone Disease.

**Methods:**

Seventy-five carers from the UK and USA participated in this cross-sectional study, between July 2021 and February 2023. Participants completed assessments on: anticipatory grief (MMCGI-SF, comprising three sub-scales: Personal Sacrifice Burden, Heartfelt Sadness and Longing, Worry and Felt Isolation); person with MND (pwMND) behavioral changes (MiND-B) and disease severity (ALSFRS-R); carer-pwMND emotional bond (Relationship Closeness Scale), familism levels (Familism Scale), and reported hours of care provided. Multiple linear regression analyses were conducted to explore factors impacting carer AG.

**Results:**

AG total scores showed that 50.7% of carers were experiencing common grieving reactions, 22.6% presented intense grieving emotions, and 26.7% presented low grieving responses.

Disease severity (regression coefficient, β = −0.31, *p* = 0.01, 95%CI −0.91 to −0.13) and behavioral changes (β = −0.34, *p* = 0.002, 95%CI −1.45 to −0.33) predicted AG total scores (proportion of explained variation, *R*^2^=0.38, *p* < 0.001).

Regarding AG subscales, Personal Sacrifice Burden (*R*^2^=0.43, *p* < 0.001) was predicted by disease severity (β = −0.39, *p* < 0.001, 95%CI −0.42 to −0.11). Behavioral changes predicted Heartfelt Sadness and Longing (β = −0.27, *p* = 0.03, 95%CI −0.49 to −0.03; *R*^2^ = 0.21, *p* = 0.01) and Worry and Felt Isolation (β = −0.42, *p* < 0.001, 95%CI −0.63 to −0.20; *R*^2^=0.33, *p* < 0.001).

**Conclusion:**

This study suggests that disease-related factors may be the strongest predictors of carer AG. Interventions addressing carers’ understanding and management of MND symptoms seem crucial to support their experiences of loss and their acceptance of MND. Evidence-based support for carers in MND services is required.

## Introduction

Motor Neurone Disease (MND) is a neurodegenerative, progressive, and terminal multisystem disease affecting not only the person living with MND (pwMND) but also their carers. People with MND are mostly cared at home by family members, who are continuously experiencing everyday lifestyle changes and losses during the progression of the disease, leading to anticipatory grief (AG) (1–3). This phenomenon has been defined as the reaction to the awareness of the impending loss of a terminally ill individual and the responses to the associated losses and changes in the past, present and future (4,5). Symptoms of AG may include sadness, anger, guilt, frustration, ambiguity, loneliness, sleep disturbances and disbelief (6) and encompass grief-related emotions which surface before death, causing emotional destabilization (7) and emotional distress (8).

Interest in investigating MND family carers’ grieving processes has grown significantly due to the need for a deeper understanding of the factors influencing grief and provide more effective emotional support to carers (9–12). However, research revealed that interventions aimed at improving MND carers’ wellbeing have not specifically addressed AG to date (13).

It is likely that AG in MND is influenced by several factors, especially because motor and behavioral symptoms are present. From a motor perspective, studies in Alzheimer’s (1) and Parkinson’s disease (14) revealed that advanced stages of the disease and severe symptoms have greater impact in carer AG. Current literature in MND suggests that longer hours of care impact on post-death grief (11) and are associated with prolonged grief disorder (PGD) (15). However, the effect of disease severity and hours of care in AG in MND is unclear. From the behavioral symptomatology perspective, behavioral changes seem to be the best predictive factor of AG in dementia (16) and a risk factor for higher levels of AG in dementia (17) and Parkinson’s (18). Thus, identifying the role of behavioral changes in MND carers’ AG seem to be crucial for future interventions.

In the exploration of AG among carers, it is also important to delve into carer-related factors, to comprehend the broader context of carer AG. The literature recognizes that changes in the carer-pwMND relationship may negatively contribute to AG emotions (9), but this finding is not universally applicable. Considering the potential role of familism in carer AG is also crucial. Familism is defined as a strong family identification and attachment, characterized by feelings of loyalty and responsibility for care delivery (19). A previous systematic review revealed that higher familism values is linked to higher anxiety and depressive symptoms in carers of people living with dementia (20), and have both positive and negative impacts on emotional distress among this carer population (21). Given this association with mental health outcomes, familism values may be related to AG in MND carers.

While awareness of AG emotions among carers has been on the rise, there remains a pressing need for in-depth exploration of this phenomenon to enhance the possibility of effectively addressing it. Based on the present evidence, this exploratory study aims to examine the predictive effects of different potential factors (disease severity, behavioral changes, relationship closeness, familism and hours of care) in MND family carers’ AG.

## Materials and methods

This cross-sectional study took place between July 2021 and February 2023. Initially, recruitment commenced in July 2021 in the United Kingdom. To increase participant numbers, the study’s information was subsequently disseminated in the United States in October 2022. Recruitment occurred through dissemination of the study information in MND/ALS Associations, carer support groups and social media (e.g. FactorMND- former Twitter). Additionally, in the UK, two specialist tertiary hospitals (Norfolk and Norwich University Hospitals and Sheffield Teaching Hospitals) disseminated the study information using leaflets, participant information sheets and social media posts.

Participants completed a survey through an online platform or in paper format. The survey was completed in the same order by all participants. Online survey data were collected and managed by the Joint Information Systems Committee electronic data. All data were collected anonymously.

### Participants

Family carers currently supporting a relative living with MND, who were 18 years or older and unpaid were included in the study. No other inclusion or exclusion criteria were considered.

### Instruments included in the study

#### Anticipatory Grief – Carer

The Marwit-Meuser Caregiver Grief Inventory-Short Form (MMCGI-SF) (22) was used to measure the grief experience in carers. The measure contains three sub-scales: Personal Sacrifice Burden, identifying individual losses carers experience due to their carer role; Heartfelt Sadness and Longing, identifying intrapersonal emotional reactions related to caregiving; and Worry and Felt Isolation, identifying carers’ feelings of losing social connections and support from others. Each of the 18 items is rated on a 5-point Likert scale ranging from 1 = Strongly Disagree to 5 = Strongly Agree. Total AG score ranges from 18 to 90. Subscores from the sub-scales range from 6 to 30. Scores in the average range are common responses to loss in the caregiving experience, high scores may indicate the need for support and low scores may mean positive coping adaptation or denial. The Cronbach’s alpha for total grief score in this study was .94.

For this study, one scale item was adapted to MND carers (with author’s permission) as the instrument was originally developed and validated with dementia carers.

#### MND disease severity

The revised version of the ALS Functional Rating Scale (ALSFRS-R) (23) was used to measure the progression of functional disability. Each of the 12 items is rated on a 4-point Likert scale ranging from 0 = no function to 4 = normal function (min 0, max 48) with lower scores indicating greater disability.

The ALSFRS-R was completed by carers. Previous research has demonstrated excellent reliability of the ALSFRS-R evaluations conducted by healthcare professionals, carers, and patients (24,25). Furthermore, the ALSFRS-R included in our study provided clear instructions for scoring, tailored to ensure accessibility to individuals without specialized medical knowledge.

#### Behavioral changes in MND

The Motor Neurone Disease Behavioral Instrument (MiND-B) (26) was used to establish the presence of apathy, disinhibition, and stereotypical behavior. Each of the nine items is rated on a 4-point Likert scale ranging from 1 = everyday to 4 = no changes from normal behavior (min 9, max 36). The cutoff score indicating presence of behavioral changes is <34; with lower scores representing greater behavioral changes.

#### Carer’s perception on relationship closeness

The Relationship Closeness Scale (RCS) (27,28) was used to detect the current quality of emotional bond between the carer and the pwMND. Each of the six items is rated on a 4-point Likert scale ranging from 1 = Strongly Disagree to 4 = Strongly Agree (min 4, max 24). Responses capture carers’ degree of agreement about their relationship with the care recipient. Higher scores are indicative of closer dyadic relationships.

#### Familism – Carer

The Familism Scale (FS) (19) was used to assess the tendency to prioritize one’s family over oneself. Each of the 14 items is rated on a 5-point Likert scale ranging from 1 = very much in disagreement to 5 = very much in agreement (min 14, max 70). Higher scores indicate higher familism values.

While initially designed for Hispanic/Latino populations, the scale demonstrated high familism values among non-Hispanics individuals (19). Moreover, it has been used in studies involving diverse carer populations and different cultures, including dementia carers, revealing the impact of familism values on carers’ emotional distress (21).

#### Demographics

Demographic information about the carer and the pwMND was collected. Information for carer included age, gender, country of residence, relationship to the pwMND, hours per week they provided care, and place of residence. Information about the pwMND included age, MND phenotype, and months since diagnosis.

### Statistical analyses

Descriptive analysis of demographic information was performed to characterize the sample.

To investigate potential factors associated with AG and assess their eligibility for inclusion in a regression model, correlational analyses were conducted with carer AG as dependent variable and potential independent variables (disease severity, behavioral changes, relationship closeness, familism and hours providing care per week). A *p* value threshold of 0.25 was used to screen potential independent variables. Participants’ country of residence was included as an adjusting factor to account for any possible difference between responses from the two countries.

Subsequently, a multiple linear regression analysis was conducted to examine to what extent different carer- and disease-related factors predicted total score of carer AG. To further understand the relation of AG and predictive factors, three separate multiple regression analyses were conducted with each of the individual subscales of the MMCGI-SF as dependent variables and the same independent variables previously mentioned. The overall model fit of each regression analysis was assessed using the *F*-test and the proportion of the explained variation by the model (*R*^2^). A *p* level < 0.05 was considered statistically significant.

The Tolerance value and variance inflation factor (VIF) were estimated to check multi-collinearity, and the visual examination of the Normal Probability Plot (P–P) and scatterplot of the regression standardized residuals against predicted values were used to check for normality, linearity, and homoscedasticity assumptions.

Data analyses were performed using IBM SPSS statistical software (version 28).

## Results

Seventy-nine participants (UK = 74; USA = 5) were recruited for the study. Four respondents partially completed some of the instruments, therefore, they were removed from the data analyses which resulted in a dataset of 75 carers (UK = 70; USA = 5). Post-hoc power calculation for the regression model with the primary outcome (anticipatory grief) shows that the sample size (*n* = 75) is slightly smaller than that required (*n* = 79) to detect the overall effect size with adequate statistical power. However, as the regression coefficients with reasonable size were highly statistically significant, sample size seemed to be adequate for detecting effects of individual predictor variables. This can be considered as a reasonable compromise given the challenging (hard to recruit) nature of the target population.

The results of each single regression analysis indicated that severity of the disease, behavioral changes, relationship closeness, familism and hours of care per week passed the screening step (*p* < 0.25) and were entered to the regression models as independent variables (Supplementary Table 1).

### Characteristics of carers and people living with MND

The majority of carers were female (65.3%), their mean age was 63.09 (SD = 10.46), they were co-habiting with (94.7%) and looking after a spouse/partner living with MND (89.3%) for an average of 38.64 months. Approximately a quarter of carers provided more than 100 hours of care/week (Table 1). Carer Relationship Closeness responses (mean = 16.61), suggested that, as a group, carers had a relatively close dyadic relationship with the care recipient. Familism responses (mean = 42.04) suggested moderate levels of familism values. (Table 2).

**Table 1.** Demographic characteristics of family carers and people living with MND (*n* = 75).

Family carer	Frequency and %, M (SD)
Age (mean, SD)	63.09 (10.46)
Gender (Female)	49 (65.3%)
Relationship to PwMND	
Spouse/Partner	67 (89.3%)
Parent	3 (4%)
Child	4 (5.4%)
Other	1 (1.3%)
Living with PwMND (yes)	71 (94.7%)
Months caring (mean, SD)*	38.64 (44.08)
Country of Residence (frequency)	
UK	70 (93.3%)
United States	5 (6.7%)
Hours providing care per week	
<1–8	17 (22.6%)
9–30	19 (25.4%)
31–49	10 (13.3%)
50–99	10 (13.3%)
100 or more	19 (25.4%)
**People living with MND**	**Percentage or M (SD)**
Age (mean, SD)	64.70 (11.74)
Gender (Male)	45 (60%)
Phenotype of MND*	
ALS	37 (49.3%)
Progressive Bulbar Palsy	9 (12%)
Progressive Muscular Atrophy	7 (9.3%)
Primary Lateral Sclerosis	8 (10.7%)
ALS-FTD	2 (2.7%)
Don’t know	11 (14.7%)
Months since diagnosis (mean, SD)	45.08 (47.88)

*Note:* *Missing data for months caring (*n* = 73/75), phenotype of MND (*n* = 74/75) and months since diagnosis (*n* = 73/75). Phenotypes of MND were reported by carers.

**Table 2.** Results from clinical variables included in the multiple regression analyses (*n* = 75).

Clinical variables frequency and % or M(SD)	
Anticipatory grief – carer (MMCGI-SH)	
Total grief score	
High grief profile	17 (22.6%)
Average grief profile	38 (50.7%)
Low grief profile	20 (26.7%)
Personal Sacrifice Burden	
High grief profile	19 (25.3%)
Average grief profile	38 (50.7%)
Low grief profile	19 (24%)
Heartfelt Sadness and Longing	
High grief profile	13 (17.3%)
Average grief profile	39 (52%)
Low grief profile	23 (30.7%)
Worry and Felt Isolation	
High grief profile	18 (24%)
Average grief profile	42 (56%)
Low grief profile	15 (20%)
Disease severity – pwMND (ALSFRS-R)	24.99 (9.11)
Behavioral changes – pwMND (MiND-B)	30.13 (5.86)
Presence of behavioral changes	49 (65.3%)
Relationship closeness – carer (RCS)	16.61 (2.47)
Familism – carer (FS)	42.04 (7.76)

People living with MND were mostly men (60%), mean age was 64.70 (SD = 11.74). On average, they had received the formal diagnosis of MND within the previous 45 months (Table 1). More than half of them (65.3%) exhibited behavioral symptoms as rated by carers in the MiND-B, highlighting the prevalence of behavioral symptomatology within this population. The mean score for the ALSFRS-R was 24.99 (Table 2).

### Carers’ experience of AG

Anticipatory grief total scores showed that approximately half of the carers (50.7%) were experiencing common grieving reactions. While 22.6% of carers had more intense grieving reactions and may have been in need of support for better coping, 26.7% of the carers presented a low grief profile, indicating either good coping with emotions or possible denial of emotions (Table 2).

**Figure 1.**
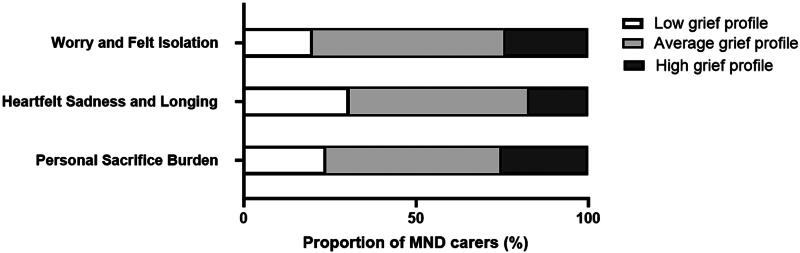
Anticipatory grief subscales based on carer self-report (MMCGI-SF).

Results from each of the subscales showed that almost half of the carers (around 50%) were transiting normal grief reactions for each of the three subscales, suggesting they were going through expected emotional responses related to their caregiving role. Furthermore, 25.3% of carers were in the high grief categories for Personal Sacrifice Burden, 17.3% for Heartfelt Sadness and Longing, and 24% for Worry and Felt Isolation. The sub-scale Heartfelt Sadness and Longing had the highest percentage of carers in the low grief profile, with 30.7% falling into this group (Table 2; Figure 1).

### Which are the factors that best predict carer AG?

Correlation results for variables included in the regression model with AG total score as dependent variable can be found in Table 3.

**Table 3.** Pearson’s *r* correlations among variables when including MMCGI-SH total score as dependent variable (*n* = 75).

Variables	1	2	3	4	5	6	7
1. Anticipatory grief (MMCGI-SH)	1.00						
2. Disease severity (ALSFRS-R)	−0.48**	1.00					
3. Behavioral changes (MiND-B)	−0.48**	0.36**	1.00				
4. Relationship Closeness (RCS)	−0.29**	0.18	0.25*	1.00			
5. Familism (FS)	−0.16	0.09	0.14	0.21*	1.00		
6. Hours of care per week	0.28**	−0.48**	−0.19	−0.00	0.03	1.00	
7. Country of residence	−0.12	−0.01	−0.11	0.15	0.18	−0.08	1.00

** *p* < 0.01.

* *p* < 0.05.

*Disease severity* and *behavioral changes* were the only two variables contributing to carer AG, and the model accounted for approximately 38% of the variance (Table 4).

**Table 4.** Factors explaining the variance of carer anticipatory grief as measured by the MMCGI-SH (total score and sub-scales scores) (*n* = 75).

	Anticipatory grief *(Total score)*			95% CI	
Predictors	B	*t*	*p*	Lower	Upper
**Disease severity**	**−0.31**	**−2.64**	**0.01**	**−0.91**	**−0.13**
**Behavioral changes**	**−0.34**	**−3.18**	**0.002**	**−1.45**	**−0.33**
Relationship closeness	−0.12	−1.21	0.23	−2.05	0.50
Familism	−0.04	−0.40	0.69	−0.48	0.32
Hours of care (weekly)	0.06	0.53	0.60	−0.91	1.58
Country of residence	−0.13	−1.28	0.21	−20.04	4.41
*F* 7.05 d.f 6 *R*² 0.38					
	Personal Sacrifice Burden			95% CI	
Predictors	β	*t*	*p*	Lower	Upper
**Disease severity**	**−0.39**	**−3.47**	**<0.001**	**−0.42**	**−0.11**
Behavioral changes	−0.20	−1.97	0.053	−0.43	0.003
Relationship closeness	−0.10	−1.00	0.32	−0.75	0.25
Familism	−0.06	−0.62	0.54	−0.20	0.11
Hours of care (weekly)	0.20	1.88	0.07	−0.03	0.94
Country of residence	−0.11	−1.10	0.28	−7.42	2.15
*F* 8.66 d.f 6 *R*² 0.43					
	Heartfelt Sadness and Longing			95% CI	
Predictors	β	t	*p*	Lower	Upper
Disease severity	−0.24	−1.82	0.07	−0.31	0.01
**Behavioral changes**	**−0.27**	**−2.27**	**0.03**	**−0.49**	**−0.03**
Relationship closeness	−0.10	−0.82	0.41	−0.74	0.31
Familism	0.01	0.11	0.92	−0.15	0.17
Hours of care (weekly)	−0.08	−0.63	0.53	−0.67	0.35
Country of residence	−0.16	−1.44	0.15	−8.65	1.39
*F* 2.94 d.f 6 *R*² 0.21					
	Worry and Felt Isolation			95% CI	
Predictors	β	*t*	*p*	Lower	Upper
Disease severity	−0.17	−1.37	0.18	−0.26	0.05
**Behavioral changes**	**−0.42**	**−3.81**	**<0.001**	**−0.63**	**−0.20**
Relationship closeness	−0.13	−1.25	0.22	−0.81	0.19
Familism	−0.05	−0.52	0.61	−0.19	0.11
Hours of care (weekly)	0.02	0.15	0.88	−0.45	0.52
Country of residence	−0.07	−0.65	0.52	−6.30	3.20
*F* 5.60 d.f 6 *R²* 0.33					

The Tolerance (>0.67) and VIF (<1.48) values for total score indicated that there is no obvious concern of multicollinearity for the independent variables. The results of the normal P–P Plot and the scatterplot of the standard residuals showed that the assumption of normality, linearity and homoscedasticity of residuals were met.

### Are disease-related factors also the best predictors of the different aspects of AG?

**Figure 2.**
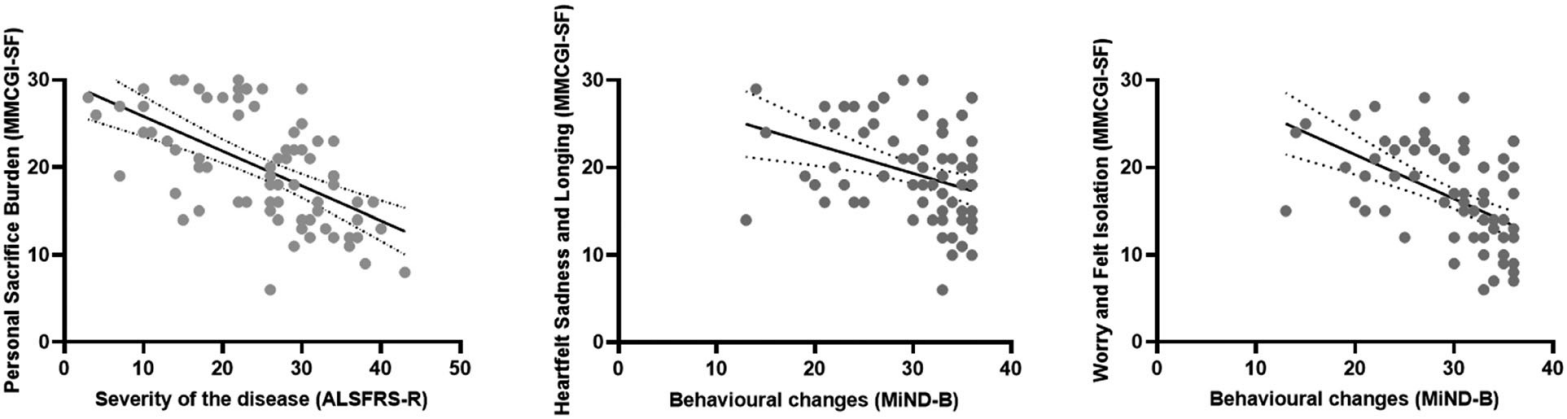
Predictive factors against each subscale from MMCGI-SF. MMCGI-SF higher scores denote higher grief emotions. MiND-B lower scores indicate more marked behavioral changes. ALSFRS-R lower scores indicate greater disability.

When analyzing individual subscales from the MMCGI-SF separately (Figure 2), results indicated that *disease severity* was the only variable predicting Personal Sacrifice Burden and explained 39% of the variance in this model (Table 4). It appears that as MND progresses, carers’ grieving emotions related to everyday changes and losses become more pronounced and carers experience increasing levels of grief.

*Behavioral changes* were the only factor significantly contributing to Heartfelt Sadness and Longing, accounting for 27% of the variance of AG. This suggests that when behavioral changes are present, carers’ intrapersonal grieving reactions, such as sadness, anger, guilt, and feelings of powerlessness are likely to emerge.

*Behavioral changes* were the only factor predicting Worry and Felt Isolation, accounting for 42% of the variance on carer AG, suggesting that carers’ feelings of losing connections and support from others tend to increase due to the presence of behavioral symptoms.

The Tolerance and VIF values for each subscale indicated that multicollinearity is not a significant concern, as all Tolerance values were greater than 0.6, and all VIF values were below 1.49. Additionally, the examination of the Normal P–P Plots and the scatterplots of the standard residuals for each subscale suggested no violations of the assumption of normality, linearity and homoscedasticity of residuals.

## Discussion

This study explored the impact of disease- (i.e. disease severity and behavioral changes) and carer-related factors (i.e. relationship closeness, familism, and hours of care) on carer anticipatory grief. Results demonstrate that disease-related factors are significant predictors of AG, but carer-related factors do not appear to have significant impact on AG. Findings suggest that behavioral changes have a strong contribution to carers’ intrapersonal feelings of changes, losses, and isolation, over and above other variables.

Behavioral changes, such as disinhibited behavior, apathy, and stereotypical behaviors, seem to be the primary predictor of AG, including the specific aspects of AG as measured by the MMCGI-SF subscale Heartfelt Sadness and Longing, i.e. intrapersonal emotions connected to traditional grief concepts. It appears that feelings of sadness, anger and guilt are strongly linked to changes in the pwMND’s behaviors, i.e. the loss of the person before behavioral symptoms presented or the difficulties in managing them. This is consistent with studies involving dementia carers, where behavioral problems were the best predictor of AG (16), and with carers of people with mild cognitive impairment which reported “missing the person” upon diagnosis (29). Additionally, challenging behaviors can strain the carer-pwMND relationship and impact AG (9). The underlying cause of relationship changes may be the behavioral symptoms. Notably, in this study, relationship closeness did not significantly impact carer AG. This may be because the study measured perceived closeness, not changes in the relationship itself.

Similarly, behavioral changes were the sole factor significantly affecting Worry and Felt Isolation subscale, which encompasses carers’ feelings of isolation and lack of support. Previous research has linked these feelings to progressive caring duties and a lack of communication with healthcare professionals, family, friends and the pwMND (30). Building upon the literature, this study identified their impact on AG. It is likely that carers experience increased isolation as difficulties arise when socializing. For example, the pwMND may exhibit disinhibited behavior toward others, and carers may reduce social contact to avoid potential embarrassment from these behaviors. Moreover, carers may not feel well supported as assessment and guidance on behavioral symptoms do not feature in most MND services (31,32). As such, these symptoms sometimes occur without acknowledgement that they are part of MND and carers may not articulate or understand the changes they observe and are not offered specialist support to manage behavioral symptoms (31).

While existing evidence has highlighted the impact of the carer-pwMND relationship, and hours of care provided on post-death and PGD in MND carers (9), this study finds that when modeled alongside disease-related factors, they do not significantly explain carer AG. Furthermore, no association was discovered between familism and AG in this study. A possible explanation for these findings may be that grieving emotions may be primarily linked to the anticipated loss of the pwMND, reflected on the progressive deterioration of the person—rather than being influenced by their previous relationship or familism values as measured by scales used, or the hours providing care. These factors may act as contextual variables that influence the intensity of grieving reactions by either exacerbating or alleviating emotions and coping, albeit not being directly linked to AG. Another possible reason might be the lack of diversity in the closeness of a dyadic relationship, and familism values, within the sample.

The present study identified disease severity as the strongest predictor of Personal Sacrifice Burden items in the MMCGI-SF, i.e., of the losses and changes carers experience due to their caring role. A systematic review revealed that some changes and losses result from carers’ lack of personal time due to increasing hours of care (30). Interestingly, our study suggests that AG might not be highly influenced by additional strain caused by hours of care, implying that other factors play a more substantial role in the perception of loss. It is plausible that AG responses are triggered as disability progresses, leading the family to mourn the person they once knew and anticipate future losses. This aligns with findings in other neurodegenerative diseases, such as dementia and Parkinson’s, where grieving emotions intensify as the disease advances (2,11).

Previous research with MND carers (8,33) highlighted the vulnerability and loneliness that carers experience, as well as the insufficient emotional and practical support services available to address their needs. Consequently, it is not surprising that in this study carers reported higher scores in the Personal Sacrifice Burden and Worry and Felt Isolation domains on the MMCGI-SF. The low *R*^2^ scores implied the presence of additional variables influencing AG. For example, certain factors considered to be protective for carer wellbeing, such as social support (34) and coping strategies have not been included in this study and should be explored in future studies. Furthermore, research has identified coping mechanisms, such as adopting a proactive approach to daily changes, focusing on positive aspects of life, and compartmentalizing negative thoughts, as effective strategies for managing everyday changes and losses due to MND, and merit further investigation (7,8).

This study has some limitations. The MMCGI-SF was used to assess carer AG, where low scores might mean that carers are in denial and not necessarily coping well and adaptively; thus, these findings need to be interpreted with caution. Moreover, carers from this study presented a close dyadic relationship, which could have influenced the findings. The absence of measures of anxiety and depression as potential confounding factors when carers completed self-rating measures on the survey could have also influenced the results. Cognition in the pwMND was not assessed in this study. Considering the prevalence of changes in cognition in this population, future studies should include and assess how cognitive symptoms may affect carer AG. Furthermore, the vast majority of carers in this study were spouses/partners of the pwMND. Future research should consider examining the AG experience of other family carers not living in the same residence, or carers who may have a different relationship with the pwMND, such as adult–children; as well as investigating AG emotions between genders. Another limitation of this study is the difference in the number of participants from UK and USA. Despite including country of residence as an adjusting factor to control for potential confounding effects of nationality on the outcome variable, the imbalance in sample sizes could affect the interpretation and generalization of results, though these are likely to be minimal given the stringent statistical approach. Future research with a more balanced distribution of participants from different countries could provide further insights into the relationships under investigation.

This study enhances our understanding of factors influencing AG. Both disease severity and behavioral changes are non-modifiable factors, posing a challenge in supporting carers’ experiences of loss. The findings underscore the need for targeted interventions to help carers cope with AG emotions. Additionally, this study emphasizes the importance of addressing and managing behavioral symptoms in MND. Providing HCPs with training and offering carers strategies to handle behaviors they find challenging could have a positive impact on AG emotions. The MiNDToolkit, a novel psychoeducational online intervention for carers (35), may be a potential route to support MND carers managing behavioral symptoms in MND.

In summary, our study highlights the significance of disease symptomatology in various domains of carer AG, including the reactions to the losses and changes due to the caring role itself (e.g. loss of personal time), the intrapersonal feelings while providing care (e.g. sadness, powerlessness, denial) and feelings of losing connections and support from others. Interventions targeting support as MND progresses and education on behavioral management could alleviate grieving symptomatology and enhance MND carers’ wellbeing.

## Supplementary Material

Supplemental Material
