# Supplementary material for: Factors affecting anticipatory grief of family carers supporting people living with Motor Neurone disease: the impact of disease symptomatology
Source: Amyotroph Lateral Scler Frontotemporal Degener. 2024 May 30;25(7-8):776–84. doi: 10.1080/21678421.2024.2359559 (PMC11523914; doi:10.1080/21678421.2024.2359559)
Supplement: Supplemental Material [file IAFD_A_2359559_SM8074.docx]

**Supplementary Table 1.**

**Potential independent variables to be included in the linear regression analysis with MMCGI-SF total score as dependent variable (n=75)**

| Potential independent variables | *p* value |
| --- | --- |
| Disease severity (ALSFRS-R) | <0.001 |
| Behavioural changes (Mind-B) | <0.001 |
| Relationship closeness (RC Scale) | 0.011 |
| Familism (Familism Scale) | 0.158 |
| Hours of caring per week | 0.015 |
